# Supplementary material for: Therapeutic Drug Monitoring of Amphotericin-B in Plasma and Peritoneal Fluid of Pediatric Patients after Liver Transplantation: A Case Series
Source: Antibiotics (Basel). 2022 May 11;11(5):640. doi: 10.3390/antibiotics11050640 (PMC9137682; doi:10.3390/antibiotics11050640)
Supplement: Supplementary file 1 [file antibiotics-11-00640-s001.zip › antibiotics-1701823-supplementary.pdf]

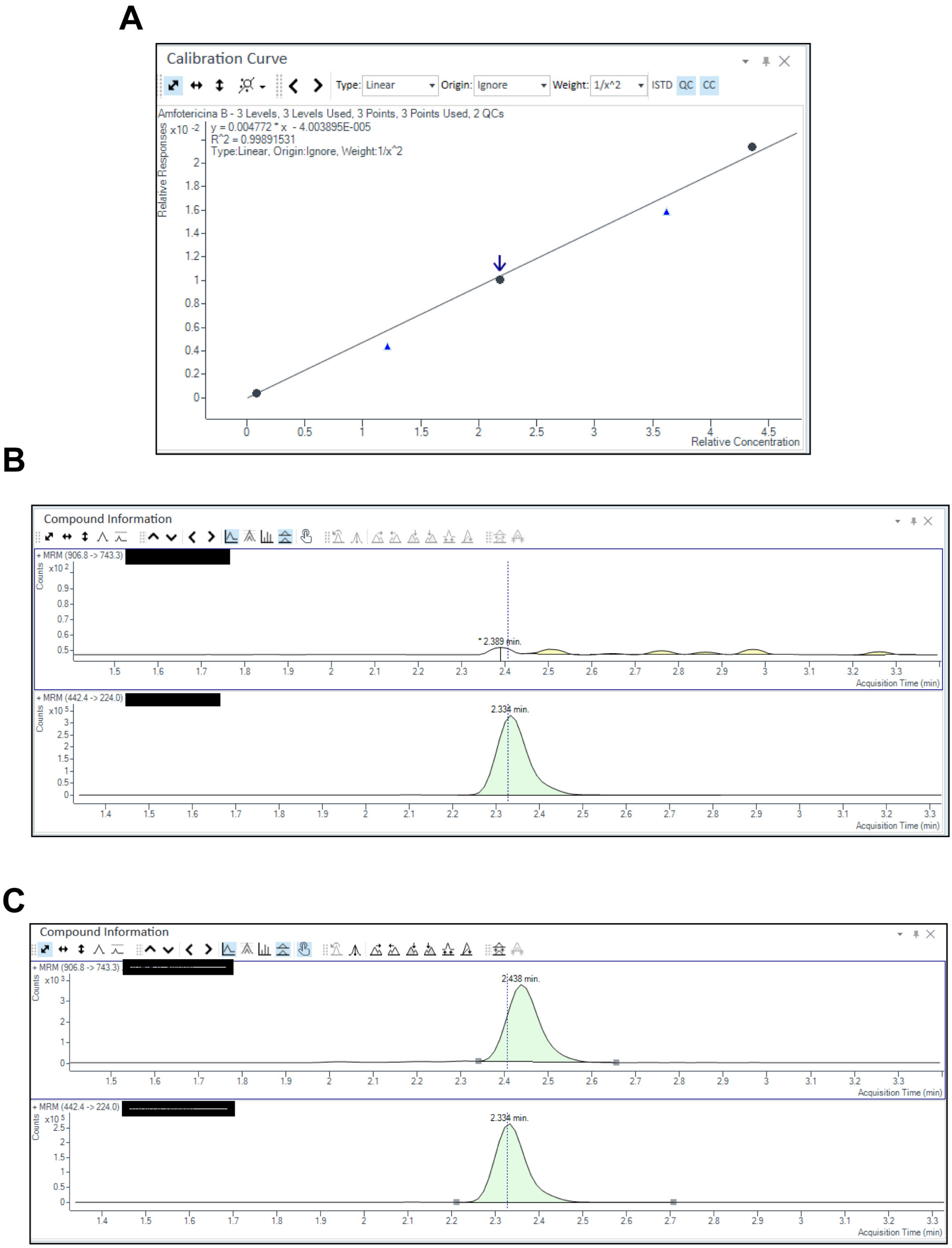

Supplementary Figure S1. Selectivity and specificity for amphotericin-B in plasma determined by LC-MS/MS. **(A)** Calibration curve for amphotericin-B. Black dots indicate calibrators, purple arrow indicates calibrator 2 (2.36 µg/mL) whilst blue triangles represent Low and High QCs. **(B)** Representative chromatogram of plasma sample from a patient treated with Isavuconazole but not amphotericin-B is

reported in the upper layer meanwhile lower layer shows the same samples spiked with an isotopically labeled internal standard (IS). **(C)** Upper layer shows a representative chromatogram of plasma sample from one patient treated with liposomal amphotericin-B and spiked with IS (lower layer). Black rectangular has been used to hide patients' identity. Retention times for amphotericin-B and IS are 2.438 and 2.334, respectively.
